# Measurement of Mood States Following Light Alcohol Consumption: Evidence from the Implicit Association Test

**DOI:** 10.3390/bs8090079

**Published:** 2018-09-03

**Authors:** Motohiro Ito, Naoyuki Matsuzaki, Jun Kawahara

**Affiliations:** 1 Department of Psychology, Faculty of Letters, Hokkaido University, N10 W7, Kita-ku, Sapporo, Hokkaido 060-0810, Japan; jkawa@let.hokudai.ac.jp; 2 Japan Society for the Promotion of Science, 5-3-1 Kojimachi, Chiyoda-ku, Tokyo 102-0083, Japan; 3 Suntory Global Innovation Center Limited, 8-1-1 Seikadai, Seika-cho, Soraku-gun, Kyoto 619-0284, Japan; Naoyuki_Matsuzaki@suntory.co.jp

**Keywords:** implicit association test, measurement of mood state, light alcohol consumption

## Abstract

As the problems of mood measurements during alcohol consumption of alcoholic beverages do not necessarily evoke interpretable physiological responses, explicit reports may be contaminated by various cognitive biases or expectations. The present study examined whether emotional responses induced by the consumption of beverages containing low concentrations of alcohol can be measured using the Implicit Association Test (IAT). The IAT can detect the estimates of internal proximity between bipolar target concepts (e.g., cheerfulness and fatigue). Participants (*N* = 30) received three IAT sessions, followed by drinking a beverage containing 0% (control), 1%, or 3% alcohol by volume, and three IATs (at 0, 30, and 60 min after the time of consumption). We also recorded the explicit responses regarding the extent of drunkenness. The analyses of variance with alcohol concentration and time reveal dissociation between implicit and explicit measures. The IAT scores under the alcohol conditions reflect a more cheerful mood state relative to the baseline test. This effect of enhanced cheerfulness was not observed under the non-alcohol control condition. These results demonstrate that the impact of the consumption of low-alcohol beverages on mood can be measured using the IAT.

## 1. Introduction

Self-report procedures are among the most commonly used methods for measuring mental states [1]. Due to their simplicity, and ease of administration and scoring, numerous questionnaires have been developed to capture a variety of mental states, intended for use with a range of participants and circumstances. To measure anxiety, for example, one can use the State-Trait Anxiety Inventory [2] and the Profile of Mood States [3]. Moreover, self-report questionnaires are also useful. For example, a population survey conducted in Australia indicated that regular drinking predicted their life satisfaction. Specifically, moderate drinkers tend to report higher life satisfaction than abstainers [4].

Similarly, various self-report procedures to be completed by participants are available for measuring mood states associated with alcohol consumption [5,6]. In a recent study, Parker et al. [7] asked participants to rate the strength of their current mood states as reflected by certain words (e.g., happy, anxious) using a visual analog scale before and after alcohol consumption. The results indicated that ratings of happiness increased, whereas ratings of anxiety decreased after alcohol consumption relative to the before-consumption baseline. Other studies also found that alcohol consumption enhanced positive mood states, as measured by a questionnaire and increased enjoyment-smiling in a social setting [8,9].

Self-report is a useful procedure for psychological assessment. However, it is not a flawless gold standard. Self-reports are susceptible to intentional alteration [10], whereby participants tend to provide responses that conform to a socially desirable norm or culture to which they belong [11,12]. It has also been shown that self-reports are subject to various cognitive biases, such as memory bias [13]. Moreover, as an assessment method used in the context of alcohol consumption, placebo and expectancy effects among drinkers have been identified as potentially unavoidable cognitive biases that may result in the conscious alteration of responses [5,6,14,15].

One way to circumvent such cognitive biases is to utilize unconscious/implicit components in measuring responses to alcohol consumption. Given that alcohol consumption is assumed to biologically trigger both implicit and explicit dimensions of responses [16], and that changes in implicit components precede explicit responses [17,18], it is reasonable to expect that implicit measures might successfully capture the changes in mental states that occur as a result of alcohol consumption, without being affected by explicit cognitive biases.

Another limitation of self-reports as an index of the effect of alcohol consumption is that extant studies have been based on experiments using beverages with high alcohol content (e.g., vodka at 37.5% alcohol by volume) [7]. It is therefore unclear whether self-reports are sensitive to changes in mood state that result from light alcohol consumption. To circumvent all of the aforementioned limitations associated with using self-reports to measure mood states, the present study measured the effects of alcohol consumption on mood states by using an objective behavioral task, known as the Implicit Association Test (IAT) [19,20].

The IAT was originally developed within the field of social psychology to estimate implicit attitudes or biases, such as those arising from sexism [21] or racism [19]. The IAT provides an estimate of internal proximity between bipolar target concepts, and is based on the assumption that categorizing similar concepts is faster/easier than categorizing dissimilar concepts. The underlying logic of the IAT is based on sematic network theory [22], that the internal proximity between concepts represented by target categories and attribute categories should be reflected in the reaction times of categorization responses. In the IAT, participants are presented with a target word, and are required to categorize it according to one of two labels assigned to the left or right side of the computer screen. For example, in one block of trials, categories related to the words “me” and “comfortable” might be assigned to the left side (self-calmness category) and categories related to “they” and “worried” might be assigned to the right side (other-anxiety category). In a second critical block, the response mapping is switched so that the left side represents self-anxiety (“me” and “worried”), whereas the right side represents other-calmness (“they” and “comfortable”) [23]. The difference between mean reaction times under the self-calmness block and those under the self-anxiety block is taken as the index (i.e., the IAT effect) reflecting the internal proximity between the target concept (anxiety) and the self. The more quickly/accurately participants are able to categorize the words in the self-anxiety block relative to those in the self-calmness block, the greater the IAT effect, with a large IAT effect reflecting a strong internal proximity between the participant’s view of themselves and anxiety.

The IAT therefore presents certain advantages over conscious self-reports, in that it offers a way to measure objective responses without contamination by the participant’s intentions. Although the IAT was originally developed to measure stable implicit attitudes (e.g., prejudices associated with gender or race), recent studies have demonstrated that the assessment tool can be used for both trait and state measurements [24]. Sato and Kawahara [23] have also reported that anxiety induced by a test anxiety manipulation can be measured using the IAT. These findings indicate that behavioral responses, such as categorization of words, are affected by internal states due to mood induction. We therefore adopted the IAT to examine changes in mood states following light alcohol consumption.

The IAT can be used an assessment tool for measuring mood states after alcohol consumption because it avoids the potential biases by participants’ intentions and expectations. For example, participants who were recruited for studies on alcohol consumption may be susceptive to demand characteristics to explicitly demonstrate the effect of alcohol. We propose that this tool may be useful for measuring the effects of light alcohol consumption, in particular, because it has been shown that changes in implicit attitudes precede explicit judgments and actions [17,18]. Hence, even if no explicit mood changes are evoked by the consumption of beverages containing low concentrations of alcohol, it is highly likely that slight changes in mood state may result in changes in objective responses, which are measurable by the IAT. We recognize that there are current claims for the theoretical attribution of the effects of the IAT [25]. To our knowledge, this is first study to measure the mood states during alcohol consumption by the IAT, and our primary intention was to obtain empirical findings on the mood state measurements during alcohol consumption using an objective behavioral task. The purpose of the present study was to examine the effects of light alcohol consumption on current mood states (e.g., cheerfulness or fatigue) using the IAT without any contamination from intentional biases, such as expectations. The participants took part in three sessions, each of which began with a baseline IAT followed by the consumption of an alcoholic or non-alcoholic beverage (at 0%, 1%, or 3% concentration), and three subsequent IATs (at 0, 30, and 60 min after the time of consumption). Previous studies found that positive emotions increased after alcohol consumption relative to the before-consumption or the control conditions [7,8,9]. Thus, we hypothesized that if the IAT is sufficiently sensitive to detect resulting mood changes, the IAT scores reflecting a cheerful mood should be higher under the alcohol consumption conditions (1% and 3% concentration) than under the non-alcohol control condition (0% concentration). In addition to capturing any changes in mood states, our goal was also to capture the time course of such changes.

## 2. Methods

### 2.1. Participants

Thirty male participants who were social consumers of alcohol (defined as 2–16 units per week based on self-report), ranging in age between 20 and 23 years (*M* = 21.4 years) were recruited from the participant pool at Chukyo University, and were paid to participate in three 2 h sessions held on separate days (total payment was ¥10,000; approximately $83). During the recruitment of participants, there were two limitations. Specifically, we did not include (1) female participants to avoid risks for drinking during pregnancy, and (2) participants younger than 20 years, due to the minimum legal age for consumption of alcohol beverages in Japan. All participants reported having normal or corrected-to-normal visual acuity and normal taste. None of the participants had a body mass index higher than 26.5. The study was approved by the institutional review board of Chukyo University. Participants were naïve to the specific purpose of the present study, and were informed at the recruiting session that the present experiment was to examine the effect of cognitive performance during alcohol consumption.

The participants visited the laboratory at least one day (Day 0) before the initial experimental session to receive instructions for the study. After providing informed consent on that day, the participants completed a form to verify that they were in good physical and psychological condition. They also received an alcohol patch test as follows. First, an experimenter placed a patch (Alcohol Tolerance Test Patch; Life Care Giken Inc., Toyama, Japan) on the inner surface of participants’ forearm for 20 min. After that, the experimenter compared the color of the skin at the patched location and the color index. These preliminary investigations were conducted with the aim of excluding individuals who had any significant current or past medical illness, or who exhibited alcohol flush reactions; however, no such participants were identified or excluded based on these criteria. Participants were required to be free of medication or illicit substances, as verified by self-report, and were required to abstain from alcohol and excessive physical activity for 24 h, from food for 3 h, and from beverages for 1 h prior to the experimental session. They were also required to be free of caffeine on the day of the experimental session. After receiving these instructions, the participants were subjected to the IAT as a practice to become familiar with the procedure at the Day 0.

### 2.2. Apparatus and Materials

At the time of each session, participants were given one of three different alcoholic or non-alcoholic drinks (350 mL of carbonated drinks at 0%, 1%, and 3% alcohol by volume). Participants were unaware of the alcohol concentrations until the debriefing after completing the entire session on the last day. Nonetheless, participants were informed at the recruiting session that they would consume beverages including light alcoholic drinks during each session, due to ethical and medical considerations. Participants were uninformed that a 0% concentration condition was included. The manipulation of the alcohol concentrations was within-subject factor. The present experiment was double-blind, so neither the participants nor the research assistants knew which beverages were provided on each session. Drinks were prepared by a third party, and all were identical in appearance and smell, containing grapefruit flavoring, and were served chilled. The order of drink consumption was counterbalanced across participants.

In the present study, we did not measure breath alcohol concentration (BrAC) or blood alcohol concentration (BAC). However, we assumed that alcohol in blood disappears 30 min after the consumption of the drink at 1% alcohol by volume. The previous studies confirmed that Japanese people can metabolize 4 g of alcohol per hour [26,27]. In this experiment, subjects consumed 2.8 or 8.4 g of alcohol during 20 min. We estimated that 1% is equivalent to 0.047 g/kg (body weight = 60 kg). In a preliminary experiment, we confirmed that the BrAC is zero after 30 min.

The IAT was administered by custom-made software presented on a 24 inch LCD monitor controlled by a PC/AT-compatible computer operating with Linux, Matlab, and Psychophysics Toolbox [28]. The viewing distance was approximately 60 cm. Responses were collected via computer mouse.

### 2.3. Implicit Association Test (IAT)

Four IATs were conducted during each experimental session: immediately before the consumption of the drink (baseline condition), immediately after consumption (0 min delay condition), 30 min after consumption (30 min delay condition), and 60 min after consumption (60 min delay condition). The manipulation of time was within-subject factor. Based on a previous study using the IAT [23], we used five Japanese words for each of the target categories, related to self, “わたし” (I, “わたしは”; my, “わたしの”; me, “わたしを”; for me “わたしに”; and with me “わたしと”) and other “あなた” (you, “あなたは”; your, “あなたの”; you, “あなたを”; for you “あなたに”; and with you “あなたと”). We also used five Japanese words for each attribute category, related to the mood states of cheerfulness, “元気” (active, “活発だ”; energetic, “活力がある”; vigorous, “元気がある”; powerful, “勢いがある”; and brisk, “爽快だ”) and fatigue, “疲労” (tired, “疲れた”; dead, “ぐったりする”; exhausted, “へとへとだ”; fatigued, “くたびれた”; and languid, “だるい”). Mood-related state words utilized in this study were extracted from a pilot study in which 34 participants (none of whom participated in the present study) evaluated the match between a specific word from a list of the Profile of Mood States (Japanese edition) [29] and alternatives provided by a thesaurus [30]. We chose the top five words in each category.

Each IAT session consisted of 7 blocks, all of which were administered in numerical order, corresponding to Table 1, in each session. A trial began with a fixation cross (0.7° in height and width) at the center of the screen, and category-label words in the upper-left and upper-right quadrants (1.2° in height and 2.3° in width) were presented 9° above, and 12.8° left or right of the center for 1000 ms. Then, a target word (1° in height and 2–3° in width) replaced the fixation cross at the center until the participant entered a response. Participants were asked to judge as quickly and accurately as possible whether the central target word was applicable to the category label in the upper-left or the one in the upper-right by clicking the corresponding button of a mouse; that is, if a target word was applicable to the upper-left category label (upper-right category label), they clicked the left (right) button on the mouse. Immediately after the response (there was no intertrial interval), the next trial was initiated, displaying a fixation cross and the category labels. The order of the target words was randomized.

In Block 1, the target words were randomly chosen from self/other category words. Participants judged whether the target word belonged to the upper-left category (always the self category) or the upper-right category (always the other category). In Blocks 2 and 5, the target words were randomly chosen from the cheerfulness/fatigue category words. Participants judged whether the target word belonged to the upper-left category (the cheerfulness category in Block 2 and the fatigue category in Block 5) or the upper-right category (the fatigue category in Block 2 and the cheerfulness category in Block 5). In Blocks 3 and 4, the target words were randomly chosen from either the self/other or cheerfulness/fatigue category words. Participants judged whether the target word belonged to the upper-left category (always the cheerfulness or self category) or the upper-right category (always the fatigue or other category). In Blocks 6 and 7, the target words were also randomly chosen from either the self/other or cheerfulness/fatigue category words. The participants judged whether the target word belonged to the upper-left category (always the fatigue or self category) or the upper-right category (always the cheerfulness or other category). Blocks 3 and 6 contained only practice trials, whereas Blocks 4 and 7 contained the experimental trials. Each IAT session took approximately 8 min to complete.

### 2.4. Procedure

**Figure 1:**
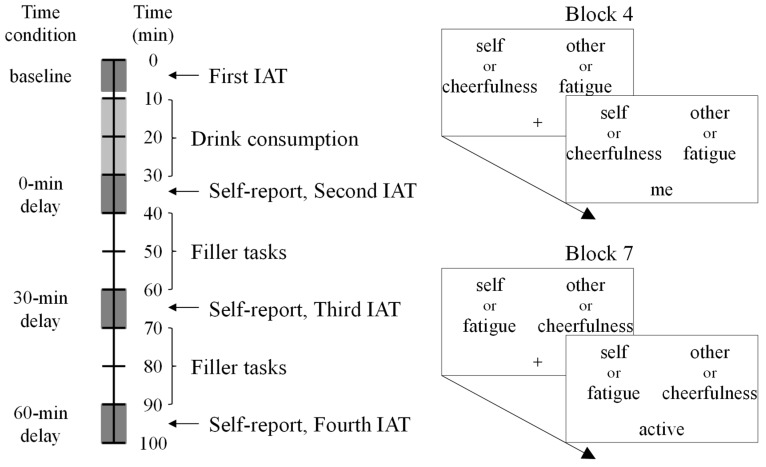
(**Left**) The sequence of events in one experimental session. The dark gray boxes represent administration of the Implicit Association Test (IAT) and the intoxication self-report. The light-gray box represents the time of consumption of the beverage. One IAT session (and one self-report pertaining to intoxication) took approximately 8 min (and 2 min) to complete. Two filler tasks were conducted during the inter-IAT periods (approximately 20 min) to avoid having participants fall asleep. (**Right**) Schematic representations of the IAT screen on the critical blocks (Blocks 4 and 7). Note that the category labels and target words in the display were shown in Japanese in the present study.

Each participant completed a total of three sessions lasting approximately 100 min each. The three sessions were distributed over a span of 9–26 days, with the constraint that two consecutive sessions were separated by at least 1 day. The three sessions were identical, except for the alcohol concentration present in the drinks consumed by the participants. Each session consisted of four IATs (see below for details) interspersed with alcohol consumption, three self-reports, and filler tasks. Figure 1 shows the sequence of events in one such session. Upon their arrival at the laboratory, the participants were welcomed by the experimenters and seated in an individual cubicle in front of a computer. After providing their consent in writing, the participants documentarily reported a form to verify that they were in good physical health. The experimenter initiated the session with a baseline IAT, immediately followed by the consumption of an alcoholic or non-alcoholic drink. The participants consumed 350 mL of the assigned drink over 20 min. Specifically, participants were provided half (175 mL) of their drink to be consumed in the first 10 min period, and received the remaining half in the second 10 min period. Immediately after consuming the drink, participants completed an IAT at each 30 min interval. The second and third IATs were directly preceded by a questionnaire that evaluated the extent of their intoxication based on seven items (intoxication, sleepiness, pleasantness, thirst, headache, light-headiness, and palpitations) to which participants responded on a scale ranging from 1 (not at all) to 9 (extremely). Participants also completed filler tasks (e.g., an operation-span task [31] and intelligence ratings of the faces in photographs) for approximately 20 min during the intervals between the completion of the IAT and the next intoxication report, to avoid having participants fall asleep. The results of the filler tasks indicated no major effect of alcohol concentration. We do not report on them further, because they were irrelevant to the purpose of the present study. After completing the third session, participants were debriefed with respect to the purpose of the study. The timing of the administration of the IATs, questionnaires, and filler tasks was controlled by the computer.

### 2.5. Data Analyses

All analyses were conducted using R version 3.3.2. Each data for reaction times of the IAT, IAT scores, or self-report scores for drunkenness was analyzed by using repeated measures analysis of variance (ANOVA), with the alcohol concentration (0%, 1%, and 3%) and time (baseline, 0, 30, and 60 min delay) as within-subjects factors. For the analysis of the reaction time data for the IAT, the critical block for the IAT (Blocks 4 and 7) was included as additional within-subject factors. For the IAT scores or the self-report scores for drunkenness, we compared these baseline scores by a one-way ANOVA with the alcohol concentration, to confirm no effect of the alcohol concentration on these indices, because the baseline data was recorded before the consumption. We also performed separate ANOVAs (see Section 3) with the time as a within-subject factor on each alcohol concentration condition. Ryan’s method *t*-tests for multiple comparison were used (*α* = 0.05).

IAT scores were calculated based on an improved scoring algorithm [20]. All reaction time data were analyzed, given that none of the participants had a response latency of less than 300 ms on more than 10% of all the trials. Our criteria required the elimination of any trials in Blocks 3, 4, 6, or 7 with a latency of greater than 10,000 ms; however, no such trials occurred in the present study. We calculated the mean latencies for correct trials in Blocks 3, 4, 6, and 7. We also calculated two additional indices: (a) the value of one standard deviation between Blocks 3 and 6, and (b) between Blocks 4 and 7. Next, the latencies for incorrect responses were replaced with the mean reaction time for the correct trials within each block + 600 ms. Subsequently, we computed the difference in latencies between Blocks 3 and 6 (and between Blocks 4 and 7), with each difference divided by the precalculated standard deviation for Blocks 3 and 6 (or Blocks 4 and 7). Finally, these two scores were averaged. These analyses resulted in a score that we deemed to reflect the IAT effect.

## 3. Results

**Figure 2:**
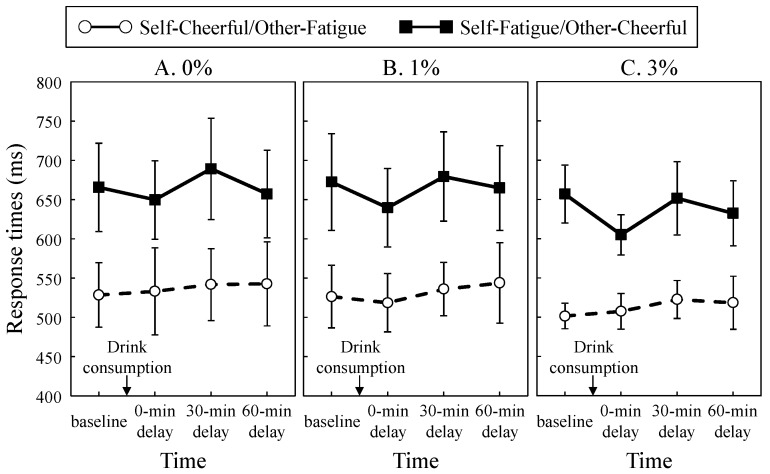
Mean reaction times of correct trials for each alcohol concentration (A: 0%, B: 1%, and C: 3%) and the two critical blocks (Blocks 4, self—cheerful/other—fatigue; and 7, self—fatigue/other—cheerful) as a function of time (baseline, 0, 30, and 60 min delay). Error bars show 95% confidence interval.

We analyzed data from only 27 participants because the remaining 3 were unable to complete the IAT sessions due to computer failure. The mean response times of the correct trials of each alcohol concentration condition and the two critical blocks (Blocks 4 and 7) as a function of time are shown in Figure 2. To compare these response times on Blocks 4 and 7 before and after consumption of the alcoholic or non-alcoholic drink, we conducted a three-way ANOVA with alcohol concentration (0%, 1%, and 3%), time (baseline, 0, 30, and 60 min delay), and critical block (self-cheerfulness on Block 4 and self-fatigue on Block 7). The main effect of time was significant (*F* (3, 78) = 4.09, *p* = 0.01, η*_p_*^2^ = 0.14). Multiple comparisons of the significant main effect of time indicated that the response times at 0 **min** of delay were shorter than those at 30 min of delay (*t* (26) = 3.65, *p* = 0.007, *r* = 0.56). We also obtained a main effect of critical block (*F* (1, 26) = 167.26, *p* < 0.001, η*_p_*^2^ = 0.87), indicating that the response times on Block 4 were shorter than those on Block 7. Neither the main effect of alcohol concentration (*F* (2, 52) = 1.25, *p* = 0.30, η*_p_*^2^ = 0.05) nor the interaction regarding alcohol concentration (*F*s < 1, *p*s > 0.68, η*_p_*^2^ < 0.01) was significant.

**Figure 3:**
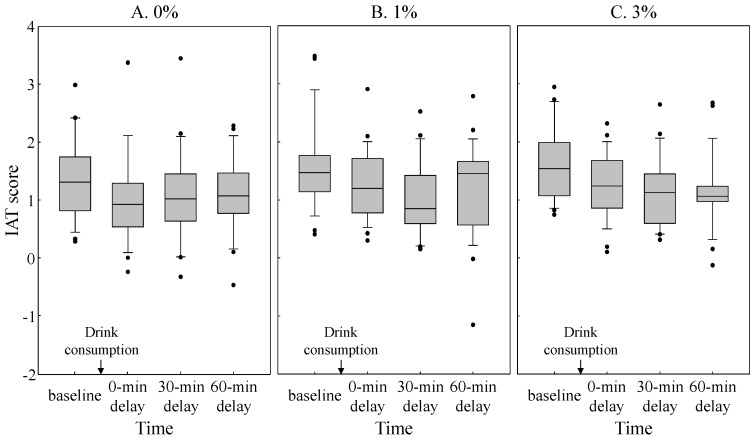
Boxplots for each of the alcohol concentrations (A: 0%, B: 1%, and C: 3%) show the median, first quartile, and third quartile of IAT scores as a function of time (baseline, 0, 30, and 60 min delay). Whiskers indicate scores to the 10th (downward) and 90th (upward) percentiles. The dot plots show outliers above (or below) the 90th (or 10th) percentile.

To examine any trends related to the IAT scores over time for each of the alcohol concentrations, boxplots were created for each alcohol concentration condition to show the median, first quartile, and third quartile of IAT scores as a function of time (Figure 3; A: 0%, B: 1%, and C: 3%). The whiskers indicate scores (downward) to the 10th and (upward) to the 90th percentiles. The dot plots show outliers above (or below) the 90th (or 10th) percentile. Regarding the interpretation of the IAT scores, positive IAT scores indicate internal proximity between fatigue and self. The IAT scores under each alcohol concentration condition showed consistently positive values over time. In the present study, as well as in other IAT studies, the absolute values of the score do not mean anything. Notably, the positive IAT scores are obtained when negative words (e.g., fatigue) were salient for participants [23]. Therefore, we focused on whether the IAT scores decreased after alcohol consumption relative to a baseline before consumption.

First, we compared the baseline IAT scores across conditions by submitting these to a one-way ANOVA with alcohol concentration (0%, 1%, and 3%) as the within-subject variable. We predicted that there would be no effect of alcohol concentration because the IAT was conducted before participants consumed the drink. The results were consistent with this prediction; the main effect of alcohol concentration was not significant (*F* (2, 52) = 1.36, *p* = 0.27, η*_p_*^2^ = 0.05). A two-way ANOVA, with alcohol concentration (0%, 1%, and 3%) and time (baseline, 0, 30, and 60 min delay) as the within-subject variables, was conducted to examine the effect of alcohol concentration on the observers’ mood states before and after consumption of the alcoholic or non-alcoholic drink. The main effect of time was significant (*F* (3, 78) = 9.01, *p* < 0.001, η*_p_*^2^ = 0.26), indicating that the IAT scores after alcohol consumption were lower than those at baseline (*t*s (78) > 3.41, *p*s < 0.005, *r*s > 0.36). Neither the main effect of alcohol concentration (*F* (2, 52) = 1.84, *p* = 0.17, η*_p_*^2^ = 0.07) nor the interaction between alcohol concentration and time (*F* (6, 156) = 0.38, *p* = 0.89, η*_p_*^2^ = 0.01) was significant.

Visual inspection of Figure 3 reveals that the median values of the IAT scores under concentration conditions of 1% and 3% (Figure 3B,C) decreased gradually over the first 30 min following consumption, whereas no such trend was found under the concentration condition of 0% (Figure 3A). This observation received statistical support in a separate ANOVA conducted for each alcohol concentration condition with time as a within-subject variable. Significant main effects of time were obtained under the concentration conditions of 1% (*F* (3, 78) = 4.15, *p* = 0.009, η*_p_*^2^ = 0.14) and 3% (*F* (3, 78) = 4.54, *p* = 0.006, η*_p_*^2^ = 0.15), but not under the concentration condition of 0% (*F* (3, 78) = 1.31, *p* = 0.28, η*_p_*^2^ = 0.05). Under the concentration condition of 1%, the IAT score at 30 min of delay was significantly lower than that under the baseline condition (*t* (78) = 3.42, *p* < 0.001, *r* = 0.36). Furthermore, under the concentration condition of 3%, IAT scores were lower at 0, 30, and 60 min of delay than they were at baseline (*t*s (78) > 2.64, *p*s < 0.01, *r*s > 0.29). These results indicate that the association between the observers’ self- and cheerfulness-concepts was apparent immediately after consumption under the concentration condition of 3%, and slightly later under the concentration condition of 1%. The association persisted longer (60 min) under the higher concentration condition (3%) than it did at the lower concentration of alcohol (1%). No such association was observed under the control (0%) condition.

Table 2 shows the mean scores for each of the seven intoxication questions over time for each of the alcohol concentration conditions. When collapsed over time, the mean scores for each of the conditions was significantly greater than “1” (representing “not at all”, 0%: *t* (26) = 8.27, *p* < 0.001, *r* = 0.85; 1%: *t* (26) = 9.50, *p* < 0.001, *r* = 0.88; 3%: *t* (26) = 8.54, *p* < 0.001, *r* = 0.86) on the intoxication scale, indicating that the participants explicitly reported that they were intoxicated regardless of the concentration of alcohol in the drink they consumed. To examine the effects of alcohol concentration on explicit reports over time, these scores were submitted to a two-way ANOVA with alcohol concentration (0%, 1%, and 3%) and time (0, 30, and 60 min delay) as the within-subject factors. The main effect of alcohol concentration was significant (*F* (2, 52) = 6.00, *p* = 0.005, η*_p_*^2^ = 0.19), indicating that participants felt more intoxicated at concentrations of 3% than they did at lower concentrations (1% and 0%) (*t*s (52) > 2.37, *p*s < 0.02, *r*s > 0.31). No difference was found between concentrations of 0% and 1% (*t* (52) = 1.00, *p* = 0.32, *r* = 0.14). The main effect of time was also significant (*F* (2, 52) = 5.05, *p* = 0.01, η*_p_*^2^ = 0.16), indicating that participants reported greater intoxication immediately after alcohol consumption than they did at 60 min post-consumption (*t* (52) = 3.17, *p* = 0.003, *r* = 0.40). No interaction between alcohol concentration and time was observed (*F* (4, 104) = 0.20, *p* = 0.94, η*_p_*^2^ = 0.008). Separate ANOVAs for each concentration condition revealed a significant effect of time only under the 0% concentration condition (*F* (2, 52) = 7.21, *p* = 0.002, η*_p_*^2^ = 0.22). The effect of time was not significant for the other concentration conditions (1%: *F* (2, 52) = 2.85, *p* = 0.067, η*_p_*^2^ = 0.10; 3%: *F* (2, 52) = 1.59, *p* = 0.21, η*_p_*^2^ = 0.06). Multiple comparisons of the significant main effect of time under the 0% concentration condition indicated that participants reported greater intoxication under the 0 min delay condition than they did under the 60 min delay condition (*t* (52) = 3.78, *p* < 0.001, *r* = 0.47). No other comparisons were significant (*t*s (52) < 2.16, *p*s > 0.035, *r*s < 0.29).

## 4. Discussion

Previous studies revealed that the positive emotions increased after the alcohol consumption on the basis of results from self-report procedures [7,8,9]. In the present study, we examined whether cheerfulness induced by light alcohol consumption could be measured by the objective behavioral task (i.e., IAT). We also recorded explicit responses for comparison purposes. The results indicated dissociation between IAT and self-report measures in two respects. First, IAT scores under the alcohol consumption conditions (at 1% and 3% concentration) reflected participants’ cheerful mood state in comparison with the baseline state. However, such changes in mood were not observed under the non-alcohol control condition (0% concentration). These results contrast sharply with the self-report measure, in which participants reported intoxication for all alcohol concentration conditions. Consistent with our findings, previous studies using both self-report and IAT indices have found that these indices do not necessarily behave in the same way [32,33], suggesting that each of these measures reveals different aspects of cognitive processing (see [34] for a review). The IAT, in particular, focuses on internal proximity between concepts and attributes in the absence of intentional responses. Therefore, the result showing that participants explicitly reported intoxication even when the beverage they consumed contained 0% alcohol reflects a cognitive bias (e.g., expectancy effect). By contrast, the IAT measure was unaffected by such an artefact.

Second, the IAT measure exhibited sensitivity to the amount of time that elapsed following the consumption of the alcoholic beverage: at the higher alcohol concentration, the lower the time since consumption, the stronger the emotional response—in other words, the response diminished over time. No effect of time was observed under the control (0% concentration) condition. These results are consistent with the fact that the consumption of the beverage containing a higher concentration of alcohol produced a corresponding change in the drinker’s cheerful mood state. However, explicit responses to alcohol consumption did not show such a trend. On the contrary, an effect of time was observed under the baseline condition, whereas no such effect was observed under the 1% or 3% alcohol conditions.

The IAT score decreased when the concentration of alcohol was fairly low (1% or 3%). This suggests that cheerfulness induced by alcohol consumption strengthened conceptual associations. However, the results of the explicit reports were uninterpretable with regard to the effects of time and its relation to alcohol concentration. Therefore, we argue that the IAT is a more useful measure than explicit questionnaires for detecting changes in cheerfulness that result from light alcohol consumption. However, it should be noted that the present results need cautious interpretation, because the two-way interaction between alcohol concentration and time did not reach significance, and are based on separate subsidiary analyses.

The approach of the present study is to measure more changeable and short-lived states, and not fixed attributes, such as sexism or racial biases, although it is known that IAT effects are relatively weak [35]. It would be difficult to obtain changes in the effect of the IAT when measuring stable attitudes. However, as demonstrated in the studies of mood congruent effects, behavioral responses and cognitive functions (e.g., short-term memory [36] and word recognition [37]) can be affected by including a mood or by temporal physiological states. We assume that this principle would also apply to similar word categorization task, as used in the present study. Consistent with a previous study [23] that IAT can detect changes in mood states caused by anxiety manipulation, the present study also found changes in cheerfulness after alcohol consumption.

Previous studies have measured mood states during and after alcohol consumption by using self-report procedures (e.g., State-Trait Anxiety Inventory [2] or eight-item mood measure [38] for negative and positive mood states, such as “sad”, “bored”, “cheerful”, and “happy”) [5,6,7,8,9]. However, self-reports may be contaminated by the conscious alteration of the participants’ responses. Given that previous studies using these methods have been based on experiments involving high alcohol concentrations, it has been unclear whether such explicit measures are sufficiently sensitive to detect mood changes at lower concentrations of alcohol. The present study clearly demonstrates that the IAT is an implicit measure that is sensitive enough to detect mood changes induced by low concentrations of alcohol.

Additional advantages of the IAT are its non-invasiveness and its efficiency in terms of time and cost. Due to technical advances in computer devices, the IAT can be administered at any time and location with relatively ease. These advantages make the IAT an attractive option for obtaining state-related responses compared with physiological or biochemical measures that require specialized equipment. For example, Zadra and Proffitt [39] effectively took advantage of an internet-based technique and collected data demonstrating that implicit associations have a circadian rhythm. The IAT also offers greater clarity than physiological and biochemical measures, given that the IAT can measure specific semantic associations between a concept (e.g., cheerfulness) and the self, whereas such a correspondence cannot be clearly interpreted based on physiological or biochemical measures (e.g., brain waves or hormonal markers).

## 5. Limitations and Conclusions

As for the limitations of the present study, our participants were restricted to male only and a narrow range of ages (20–23 years old). Also, the sample size in the present study was smaller than that in a related study [e.g., 7; *N* = 84] that examined the effect of alcohol consumption on mood states. These limitations prevent the generalization of the present results. To our knowledge, this is first study to detect the mood changes during alcohol consumption by using an IAT. That being said, it should be noted that future studies are to further explore the IAT in measuring mood states when drinking, because the IAT has not been a validated method to measure drinking-related expectancies (e.g., mood changes). In conclusion, we have demonstrated that the impact on mood of consuming a beverage containing a low concentration of alcohol may be measured using the IAT. In contrast to extant studies that have utilized high alcohol beverages (such as vodka at 37.5% alcohol by volume). We found that the IAT may be useful for measuring subtle emotional responses elicited by low concentrations of alcohol. The IAT also may be a more sensitive measure to detect subtle emotional responses compared to using explicit measures such as questionnaires. The present results further reveal a possibility to measure emotional states over time without any contamination from intentional biases. Thus, the present study suggests that the IAT may be useful in a variety of situations requiring the evaluation of subtle emotional states and their temporal changes in response to small amounts of consumption of alcohol, and possibly food, nicotine, or caffeine. At least, future research should obtain more empirical findings regarding interaction between the IAT and alcohol consumption.

## Figures and Tables

**Table 1:** Number of trials, target categories, and corresponding button mapping in each block. Participants categorized, as quickly and as accurately as possible, whether the central target word belongs to the category label in the upper-left or the one in the upper-right by clicking the corresponding button of a mouse. If a target word was applicable to the upper-left category label (upper-right category label), they clicked the left (right) button on the mouse.

		Category Labels		
Block	Number of Trials	Target Categories	Left Button	Right Button
1	20	Self or Other	Self	Other
2	20	Cheerfulness or Fatigue	Cheerfulness	Fatigue
3	20	Self or Other Cheerfulness or Fatigue	Self Cheerfulness	Other Fatigue
4	40	Self or Other Cheerfulness or Fatigue	Self Cheerfulness	Other Fatigue
5	20	Fatigue or Cheerfulness	Fatigue	Cheerfulness
6	20	Self or Other Fatigue or Cheerfulness	Self Fatigue	Other Cheerfulness
7	40	Self or Other Fatigue or Cheerfulness	Self Fatigue	Other Cheerfulness

**Table 2:** The means (standard deviation) of self-report scores for intoxication under each alcohol concentration over time.

		Time Elapsed Since Alcohol Consumption		
		0 min	30 min	60 min
**Alcohol Concentration**	**0%**	2.61 (1.02)	2.42 (0.90)	2.17 (0.92)
	**1%**	2.72 (1.06)	2.53 (0.95)	2.37 (0.84)
	**3%**	3.01 (1.43)	2.89 (1.23)	2.69 (1.08)
